# Appeal to fear in health care: appropriate or inappropriate?

**DOI:** 10.1186/s12998-017-0157-8

**Published:** 2017-09-20

**Authors:** J. Keith Simpson

**Affiliations:** Discipline of Chiropractic, Murdoch University, South Street, Murdoch, WA 6150 Australia

**Keywords:** Health care marketing, Appeal to fear, Appeal to fear fallacy, Chiropractic subluxation

## Abstract

**Aim:**

This paper examines appeal to fear in general: its perceived positive aspects, its negative characteristics, its appropriate as well as its fallacious use.

**Background:**

Appeal to fear is a commonly used marketing method that attempts to change behaviour by creating anxiety in those receiving a fearful message. It is regularly used in public health initiatives such as anti-smoking, anti-drunk driving campaigns as well as in hypertension awareness campaigns. Some chiropractors appear to use appeal to fear to promote subluxation awareness and thereby encourage the use of chiropractic treatment.

Research supporting its use is equivocal; nevertheless, when used judiciously, appeal to fear probably has sufficient strengths to warrant its continued conditional use. When used to promote care for which there is no supporting evidence, its use is fallacious.

**Discussion:**

Appeal to fear has been used in health promotion campaigns for sixty years or more with the intent of modifying behaviours. While there is evidence to suggest that appeal to fear may motivate some individuals to modify offending behaviour or adopt recommended behaviour there is growing resistance to the use of appeal to fear on ethical and psychological grounds. Using appeal to fear as a tool of persuasion can be valid or fallacious depending on the truth of the premises within the argument.

When used to raise awareness about genuine health concerns such as smoking, drunk driving and hypertension appeal to fear is considered to be a valid approach with certain caveats. However, when appeal to fear, not based on evidence or reason, is used as motivator to get others to accept unnecessary interventions for unproven disorders, the use of appeal to fear is fallacious.

**Conclusion:**

In spite of the evidence against its use, it seems likely that appeal to fear will continue to be used in conjunction with other public awareness initiatives to modify recognized detrimental behaviours such as smoking and drunk driving as well as silent killers such as hypertension. However, when used to promote a treatment that has no evidentiary basis such as subluxation based practice in chiropractic the appeal to fear is a fallacy and must be stopped.

## Background: Believe me or else bad things will happen

Marketers use a variety of methods in an effort to persuade target audiences to modify behaviours in the interests of bettering their health. One of the ploys used by the developers of public health campaigns is to provoke an emotional response. Emotional appeals may include fear of ill health, death, disfigurement, as well as shame, scorn, humiliation or even disgust [1]. According to Witte [2] ^p.329^ “Fear appeals are persuasive messages designed to scare people by describing the terrible things that will happen to them if they do not do what the message recommends.” Appeal to fear is often presented as a warning:

X is something to fear; therefore Y should be implemented to prevent X.

## Examples of appeal to fear

1) Smoking kills in many ways … quit smoking right now!

X (death) is something to fear; therefore Y (stopping smoking) should be implemented to prevent X.

2) Autism is a potentially devastating neurological disorder that can be caused by vaccinations. Protect your child from autism, do not vaccinate.

X (Autism) is something to fear; therefore Y (not vaccinating children) should be implemented to prevent X.

An appeal to fear in an argument can be valid or fallacious depending on whether the facts (premises) presented are true or false. The fallacy occurs when the level of fear created does not relate to the truth of the claim [3, 4].

In example 1 the facts are true (see below) rendering the argument valid: smoking does kill and stopping smoking does prevent smoking related deaths.

In example 2, the data do not support the premises rendering the argument invalid and making this appeal to fear fallacious.

The autism-vaccine link began in 1998 with the publication of a descriptive study by Wakefield et al. [5] hypothesising a connection between the development of autism and the 8 children in the study having received a Measles-Mumps-Rubella vaccination (MMR) one month previously. In spite of Wakefield et al.’s paper providing no data supporting a causal association between MMR vaccine and autism and a lack of a plausible biological mechanism a world-wide fear emerged [6]. Even though the Wakefield et al. paper was retracted by The Lancet [7], the fear persisted prompting significant well-designed research projects to test the vaccine-autism hypothesis. The scientific evidence is clear -- there is no link between vaccines in any form and autism [8–12] and any appeal to fear related to vaccination and autism is, by definition, fallacious.

As a motivational technique, appeal to fear has a certain intuitive appeal and is by no means new. According to Walton [13], Aristotle described appeal to fear as a fundamental rhetorical strategy and it has been a commonly used strategy in marketing and politics for six decades in the modern era [14]. Appeal to fear has many aliases, including argument from adverse consequences, scare tactics and threat appeals. It is generally perceived by health promoters and the general public as an effective device for changing attitudes and behaviours with little backfire effect. [15]. Backfire effect is the phrase coined by Nyhan and Reifler to describe the apparent psychological phenomenon whereby when an unsubstantiated belief is corrected by presenting data, instead of the opinion or belief changing, it becomes further strengthened [16].

While there is a perception of little backfire effect, the research into fear campaigns indicates that they may indeed backfire leading target audiences to increase engagement in the harmful behaviors [17–21] suggesting that other options for prompting behaviour change are more appealing [14, 22, 23]. Further, there is a growing number of authors suggesting that appeal to fear needs to be examined in light of its negative ethical and psychological implications [24–26].

Notwithstanding the negative findings, appeal to fear is considered to be a legitimate, if not caveat laden, method to employ in public health campaigns [27].

The appeal to fear fallacy occurs when baseless fear is employed in an excessive or exaggerated way to persuade others to accept a concept or adopt a behaviour [3, 4]. When used in this manner appeal to fear becomes fallacious scaremongering and is a particularly deceptive misrepresentation when the level of fear created does not relate to the truth of the claim.

This commentary provides an overview of appeal to fear and its effect on consumer behaviour. It then will consider the appropriateness or otherwise of using appeal to fear within the realm of mainstream health care by looking at four common usages:

- Anti-smoking campaigns^1^
- Anti-alcohol & drunk^2^ driving campaigns
- Hypertension awareness campaigns
- Chiropractic anti-subluxation advertising

## Appeal to fear: Overview

Use of the appeal to fear, in the form of threatening health messages, is commonly used as a strategy for changing behaviours within a population in relation to public health initiatives. The tactic involves using images or messages to elicit negative emotions such as anxiety in the expectation that the audience will be motivated to adopt the healthier behaviours [28].

Several examples exist within North America and Australia with anti-smoking and anti-alcohol campaigns being obvious ones with hypertension awareness campaigns being lesser known but widely used. Appeal to fear smoking campaigns have employed everything from graphic images of the lung damage caused by smoking to appealing to the smoker’s children with varying degrees of success [29].

Early research into appeal to fear pointed to a use with caution approach and little has changed in the intervening decades. In 1953 Janis and Feshbach’s seminal research identified three main types of emotional reactions to anxiety-arousing topics.

1. Inattentiveness – a defensive tendency to avoid thoughts related to the topic.
2. Aggressiveness – a defensive tendency to become aggressive towards the communicator, likely in the form of rejecting the arguments.
3. Defensive avoidances – attempts to ward off exposure to the anxiety causing communication typically in the form of failing to recall the message, losing interest in the topic, denying or minimizing the importance of the threat [30].

Janis and Feshbach suggested a negative association between fear producing messages and intended results. They found that a message that induced a minimal amount of fear was more effective than one evoking a high fear response in terms of both positive attitude change and resistance to subsequent attitudinal retrogression [30].

In an effort to better understand the relationship between fear inducing messages and persuasion, research continued throughout the 1960’s and by end of the decade a clearer appreciation had been formulated. In short, fear appeared to be positively related to persuasion, but not necessarily on behaviour change. Rather, high fear produced greater intentions to act than did low fear, but did not result in changes in behaviour and messages delivered by highly credible sources resulted in a more immediate attitude change than messages delivered by sources with low credibility [23].

Seven theories have been put forward to explain fear appeals in behaviour change: The linear model of fear appeals the curvilinear model of fear appeals [31], the health belief model [32], the parallel process model [33], the extended parallel process model [34], the stage model [35], and the elaboration likelihood model [36]. It is beyond the scope of this paper to review them. For those interested, Ruiter et al. provide a comprehensive review of the state of the art in their 2014 paper [20].

The theories about fear appeals have focused on either the content of the message, the nature of the behavior recommended by the communication, or the characteristics of the audience receiving the message. However, all three of these aspects (message, behavior, and audience) are important and focusing on a singular aspect appears likely to yield ambiguous results. There does appear to be agreement that fear appeals are more effective when they involve health related behaviours, include an efficacy message which assures message recipients that they are capable of performing the fear appeal’s recommended actions (self-efficacy) and/or that performing the recommended actions will result in desirable consequences [21]. However, fear-arousing messages may actually have deleterious consequences for recipients with low self-efficacy, as the message may strengthen the recipient’s belief that they cannot avoid health threats [37].

Tannembaum et al.’s 2015 meta-analysis provides perhaps the best, if not controversial summary of appeal to fear research to date:

Overall, we conclude that (a) fear appeals are effective at positively influencing attitude, intentions, and behaviors; (b) there are very few circumstances under which they are not effective; and (c) there are no identified circumstances under which they backfire and lead to undesirable outcomes [38] ^p.1178^.

This is a curious finding when most of the literature suggests when members of the target audience feel threatened but they are not convinced of their self-efficacy or of the effectiveness of the alternative behaviour the resulting behaviour may be defensive, more oriented toward avoidance of the anti-smoking fear message than action to quit smoking [19, 21, 35, 39, 40]. Furthermore, messages that arouse extreme fear may cause people to deny cancer risks and may inadvertently result in more smoking [41]. Perhaps Tannembaum et al.’s interpretation of the data relates to their combining various outcomes, − attitudes, intentions and behaviors - into one single effect size, and failure to report on studies with behavioral outcomes separately. Also, they compare a high fear condition with a condition designed to depict a lower level of fear or no fear instead of with alternative behavior change methods. In short, their work neither contains convincing arguments that fear appeals change actual behavior, nor that fear appeals would be more effective than an approach based on self-efficacy in combination with risk information.

So where does this leave us? As a marketing ploy appeal to fear has an intuitive appeal but paradoxically the outcomes are often ineffective or counterproductive [42] rendering it at best an equivocal tool for changing behaviours. In spite of this, appeal to fear continues to be widely and persistently used throughout the world [28]. There is general agreement that appeal to fear when used in this manner – to motivate individuals to adopt positive public health behaviours – is not a fallacy, but rather an appropriate positive rationally motivated form of argument [28, 43]. That said, use of fear in this manner must be approached cautiously and used judiciously [28, 44]. While some have argued that from a utilitarian perspective appeal to fear campaigns are ethical [45, 46] perhaps appeal to fear should be avoided altogether given the negative consequences highlighted above. Instead, health promoters should identify effective alternatives to fear arousal by carefully developing theory and evidence-based programs as outlined by Kok et al. [17].

If appeal to fear must be used, Witte and Allen provide sage advice:

Fear appeals motivate attitude, intention, and behaviour changes—especially fear appeals accompanied by high-efficacy messages. Therefore, they can be quite useful to practitioners. However, fear appeals should be used cautiously, since they may backfire if target audiences do not believe they are able to effectively avert a threat [21] p. 605.

Some consideration needs to be given to special populations which are seen as more vulnerable to appeal to fear, specifically the elderly. Researchers have questioned the ethics of using appeal to fear marketing targeting the elderly, especially campaigns targeting health care initiatives given that there may be detrimental psychological reactions to fear provoking messages. The evidence available suggests however that the elderly are no more or less vulnerable to this type of advertising and may well be more discerning than their younger counterparts [46] and thus less likely to be affected positively or negatively.

## Anti-smoking campaigns

Cigarettes or more correctly tobacco falls into a unique category amongst consumer products. On the one hand it is highly addictive and on the other hand it kills about 50 % of long-term users – a quarter while still in middle age [47]. Doll et al.*’s* fascinating prospective study of smoking and death among British doctors began in 1951 when the ill-effects of smoking first became known and continued for 50 years. The researchers confirmed the association between smoking habits and many severe diseases (vascular, neoplastic and respiratory diseases) and the suspected relationship between smoking and 12 types of cancer as well as increased mortality rate amongst all smokers. It clearly demonstrated that stopping smoking at any age brings with it an increase in life expectancy: stopping at age 60, 50, 40, or 30 gains, respectively, about 3, 6, 9, or 10 years of life expectancy. Doll et al. chose British doctors for pragmatic reasons. One could speculate also that Doll et al.’s study group, highly educated doctors who continued to smoke in the face of building evidence regarding its ill effects, is illustrative of the highly addictive nature of tobacco. This is borne out by La Torre et al.’s study examining knowledge, attitudes, and smoking behaviours among physicians specializing in public health [48].

Doll et al.*’s* research was also instrumental in motivating governments to develop and run anti-smoking campaigns aimed to decrease the numbers of people starting to smoke and to increase those stopping the habit. The campaigns are multimodal and include print, televised anti-smoking campaigns as well as graphic images and warning text messages on cigarette packaging.

Research into the impact of graphic and text messages points in the direction of motivating smokers towards cessation activity. Larger warnings are more effective than smaller ones and graphic images are more effective than printed warnings; strengthening pack warnings is associated with increased knowledge about the risks of smoking, increased Quitline calls, reduced smoking consumption, increased quit attempts, increased short-term smoking cessation, and reduced smoking prevalence [49–52].

While such approaches do appear to have a positive impact on smoking cessation or at least intent to stop, there are unwanted side effects. According to Hackley and Kitchen appeal to fear as found in anti-smoking campaigns violates ethical and social justice norms [53]. Further, Hastings et al. alert readers to the real possibility of collateral damage amongst non-smoking viewers caused by appeal to fear in anti-smoking advertising [37].

Whether the benefits outweigh the side effects of appeal to fear in anti-smoking campaigns is up for debate. Indeed, the courts in the USA are having such a debate with notable disagreement on the legality of using appeal to fear in the face of poor evidence that such advertising would directly cause a decrease in smoking [52]. The very fact that there is controversy reinforces the earlier statement: use of fear in this manner must be approached cautiously and used judiciously [29].

## Anti-alcohol and drunk driving campaigns

Recent anti-alcohol and drunk driving campaigns in Australia have promoted the messages: ‘If you drink then drive, you’re a bloody idiot’; ‘the faster you go, the bigger the mess’ because alcohol is the main cause of Australian road deaths, accounting for a third of all road fatalities.

Over 70% of people with serious alcohol-related road injuries are male, while only 56% of people with non-alcohol-related road injuries were male. The average age of alcohol-related crash victims was 27.5 years with over 50% aged between 15 and 24 years. This is in contrast to the average age of 37.6 years for non-alcohol-related road injuries [54, 55]. In other words, teenage drivers, in particular teenage male drivers are an at risk group and thus the group whose behaviour society would most like to change [56].

In an effort to reduce alcohol related road fatality rates governments have funded and implemented targeted measures, including speed cameras, advanced laser speed detectors and compulsory breath testing. The major component from a budgetary perspective was, and continues to be, television advertisements focused on drunk driving and speed related themes, many of which include appeal to fear messages in the form of graphic images. The State of Victoria in Australia has been running this type of campaign since 1989 with research examining the effectiveness of the Victorian initiative clearly demonstrating an association between levels of publicity supporting the speed and alcohol enforcement programs and reductions in casualty crashes when other major factors are held constant: between 1989 and 1992 there was a reduction of nearly 50% in road fatalities and 40% in serious injuries [57].

Overall however, research into the effectiveness of appeal to fear campaigns for modifying drunk driving has yielded mixed results [58]. For example, Macpherson and Lewis examined drunk driving offense rates and were unable to provide any evidence supporting the hypothesis that such advertising modified drunk driving behaviour [59], while Tay (1999) attributed a significant reduction in drunk driving behaviour [60] and alcohol related road fatalities [61] to the New Zealand advertising campaign.

Given such divergent findings, Tay and Ozanne undertook a study to examine the effect of the New Zealand advertising campaign among different subdivisions of New Zealand drivers [62]. They hypothesized that appeal to fear advertising would be effective, but only among some segments of the population. Their findings supported this hypothesis. Tay and Ozanne found that fatal accident rates had been reduced among three groups of drivers: female drivers aged between 15 and 24, female drivers aged between 25 and 34, and male drivers aged between 35 and 54. However, the fatal crash rate of the main target audience of the campaign, young male drivers, remained unaffected.

Thus we are drawn to the conclusion that used prudently appeal to fear campaigns to reduce drunk driving and alcohol related fatalities may or may not be effective for the overall population and are not effective in those most at risk: teenage male drivers.

## Beware of silent killers

### Appeal to fear in public awareness campaigns for hypertension (high blood pressure) – The silent killer

Up to this point I have considered public awareness campaigns aimed at modifying behaviours such as tobacco smoking and drunk driving. It is worth considering a campaign designed to raise awareness about a medical condition (hypertension) and motivate the target audience to take action (have their blood pressure checked).

Reports by the American Heart Association (AHA) and the Centers for Disease Control and Prevention [63, 64] paint a clear picture why hypertension is a primary public health concern.

- About 1 in 3 American adults or 75 million (29%) have high blood pressure.
- Only about half (54%) of people with high blood pressure have their condition under control.
- Nearly 1 of 3 American adults has prehypertension—blood pressure numbers that are higher than normal, but not yet in the high blood pressure range.
- More than **360,000** American deaths in 2013 (almost 1000 deaths per day) included high blood pressure as a primary or contributing cause [64].

High blood pressure increases risk for dangerous health conditions:

- First heart attack: About 7 of every 10 people having their first heart attack have high blood pressure.
- First stroke: About 8 of every 10 people having their first stroke have high blood pressure.
- Chronic (long lasting) heart failure: About 7 of every 10 people with chronic heart failure have high blood pressure.
- High blood pressure is a major risk factor for kidney disease [63].

Hypertension is a lifestyle modifiable, preventable, and controllable risk described by the World Health Organisation as The Silent Killer [65]. As detailed in the AHA report, hypertension is the primary and most common risk factor for heart disease, stroke and renal diseases. It is labeled the silent killer because hypertension has no symptoms therefore the only way to determine the presence of hypertension is by measuring one’s blood pressure.

Hypertension is not confined to the developed world. Indeed, it is considered to be a worldwide epidemic and is the leading risk factor for death and disability globally. It is estimated that nearly one billion people are affected by hypertension worldwide, and this figure is predicted to increase to 1.5 billion by 2025. Nearly one-half of this population is unaware of their condition [65]. Thus, public awareness campaigns are designed to alert the target audience to the importance of

1. Having their blood pressure checked
2. Modifying lifestyle factors (diet, exercise, alcohol consumption)
3. Receiving medical attention and
4. Adhering to medical advice, including taking prescribed medication.

The World Hypertension League (WHL) is an international, nonprofit organization with membership from 85 countries; it works through its member countries to promote hypertension awareness, early detection, and the prevention and control of this modern epidemic. In 2005, in an attempt to improve the awareness of high blood pressure, the WHL initiated World Hypertension Day (WHD). The aim of WHD is raising public awareness about hypertension, its life-threatening complications and measures to prevent it. Appeal to fear plays a role in raising awareness by alerting the target audience (all adults) to the complications of hypertension: heart attack, stroke, kidney disease, and death. WHD is considered to be highly successful and is being expanded on a yearly basis [65–67].

### Subluxation: The other silent killer – Appeal to fear in chiropractic subluxation advertising

Thus far I have reviewed appeal to fear used in anti-smoking campaigns, driver safety and hypertension awareness campaigns. As discussed, to use appeal to fear in this manner is considered appropriate but its use may need to be reconsidered in terms of effectiveness and ethicality and collateral damage.

It is now appropriate to examine fallacious use of appeal to fear using chiropractic advertising as an example.

The putative lesion that chiropractors ‘treat’ is commonly referred to as chiropractic subluxation, vertebral subluxation complex (VSC) or simply ‘subluxation’ [differentiating the chiropractic lesion from orthopaedic subluxation]. Broadly speaking within chiropractic today the term ‘subluxation’ has two meanings: the Traditional Palmerian Subluxation (TPS), whereby the presence of subluxations leads to ill health and the second: The Modern Subluxation, whereby subluxations have a local biomechanical effect.

The TPS is the focus of this discussion. DD Palmer, who ‘discovered’ chiropractic in 1895 proposed that 95% of all disease (dis-ease) was due to subluxations of the spine and that the remaining 5% was caused by subluxations of the extremities, particularly the joints of the feet [68]. DD and his son BJ Palmer hypothesized that the chiropractic *vertebral subluxation* differed from the medical “subluxation” in that it interfered with the transmission of Innate Intelligence (a fraction of Universal Intelligence) [69], independent of what has come to be recognized as the action potential. However, Innate Intelligence remains a metaphysical construct [70].

The more contemporary position is that Palmerian subluxations cause interference within the nervous system, which leads to suboptimal health and symptomatic dis-ease, and that chiropractic health care is primarily involved in the detection and removal of subluxations [71]. Chiropractors who still adhere to the founder’s paradigm – and there are many such practitioners – depict and market subluxation as an enemy that needs to be contained and eliminated.

Before examining examples of subluxation marketing, it is cogent to briefly discuss the history of chiropractic and its embracing of marketing to attract clientele. Chiropractic emerged in the late nineteenth century and, in spite of fierce opposition, grew rapidly in practitioner numbers and patient numbers during the first half of the twentieth century [72]. This is in part attributable to chiropractic adopting a unique professionalism. Unlike traditional professionalism wherein altruism is a central tenet [73], chiropractic professionalism favoured entrepreneurialism with recognition of financial success as a strong indicator for accomplishment [74]. While medical professionalism witnessed an ethical ban on advertising as early as 1847 [75], the emerging chiropractic profession embraced marketing. In fact, even chiropractic students were encouraged to advertise heavily [74, 76]. The Developer of Chiropractic, BJ Palmer advised his many readers:

What you want to keep out of a news- paper is news.

What you want to get into a newspaper is an advertisement [74] p. 44.

Whether or not current chiropractic advertising is a legacy from the early days of chiropractic is moot. It is evident however that advertising claims made by some chiropractors and indeed by some chiropractic colleges are suspect [77, 78]. A few examples of subluxation marketing are in order.

WH Koch, a chiropractic author makes the danger posed by subluxation abundantly clear:

We must now look at this condition of 'disease’, Vertebral Subluxation Complex, which is so disturbing to the flow of life force and innate expression in your body that it causes pain and the progressive breakdown of the body tissue known as pathology. The V.S.C. is the silent killer, one of the most serious health threats known to man [79] p. 34.

The World Chiropractic Alliance (WCA), is a worldwide organisation promoting subluxation based care. The WCA represents the interests of more than 70,000 doctors of chiropractic and chiropractic students, In 2006 the WCA issued a position paper stating that “chiropractic care to detect and correct vertebral subluxations offers benefits for all people, including those who do not demonstrate symptoms of a disease or health condition”.

‘Patient education’ materials prepared by Chiropracticis aims to educate patients to “believe choosing to be checked for the presence of vertebral subluxation throughout a lifetime is the only logical choice”. Once patients understand 5 facts about their health, they are well on their way to being chiropractic patients for life. The five facts are:

1. Your **Spinal Cord** is located in the **spine** and is your **Nervous System’s** information highway.
2. Your **Nervous System** controls and coordinates **every** part of your body.
3. Misalignments of your spine can **DISTORT nerve messages** and contribute to a wide range of health problems.
4. Misalignments of the spine are known as **vertebral subluxations** and negatively affect your **nervous system, health** and **well-being.**
5. Maintaining the health of your spine and nervous system should be a regular part of a health lifestyle and can dramatically improve your quality of life.

Koren Publication’s What is a Subluxation? brochure tells readers that chiropractic has become so popular because “chiropractors are the only professionals trained to locate and remove subluxations. If subluxations are in your body, you cannot be truly healthy”.

JC Smith, a prolific chiropractic author and subluxation advocate advises in his paper “Killer Subluxation”.

“The idea of sudden cardiac arrest from a vertebral subluxation is just too far outside the consciousness of most people … [however] "killer subluxations” actually may not be that far from the truth.

The subluxation brochure produced by Patient Media, Inc., is perhaps the most alarmist publication. Its Subluxation pamphlet provides a succinct description of how vertebral subluxations operate as well as the importance of their detection and removal.

Maybe you’ve never heard of subluxations. That’s OK. There probably was a time when you’d never heard of AIDS or Alzheimer’s.

There are three basic causes of subluxations. Physical causes include slips and falls, accidents, repetitive motions and improper lifting. Stress, anger and fear are examples of emotional causes of subluxations. Alcohol, drugs, pollution and poor diet can be chemical causes of subluxations. The result is the vertebral subluxation complex, or more simply, a subluxation.

Subluxations are serious!

However, the most important aspect of a subluxation is its effect on your nervous system. Compromising the way your nervous system controls and regulates your body can have grave consequences. Distorted communications between your brain and your body can cause all kinds of health problems beyond just headaches and backaches.

You can have subluxations and not even know it. Like tooth decay or cancer, subluxations can be present before any warning signs appear. The results of a thorough examination can show the location and severity of any subluxations you may have.

In an essay entitled ‘If You Don’t Know Subluxation, You Don’t Know Chiropractic’ Dr. Carey N. Pabouet-Sigafoose alerts readers to the dangers of subluxations:

There’s loss of motion in the body causing death because of a subluxation. It’s a major problem, a major killer, a major cause of disease in man and we’ve got to understand that. We’ve got to take ourselves in and give it the time and the space and the effort to get these things cleared up, not in just you, but your friends and your family. It’s imperative.

These are but a select but indicative sampling of the 176,000 results obtained by conducting a Google search using ‘subluxations kill’.

Chiropractors adhering to the TPS theory or its contemporary interpretation believe that subluxations have an effect on the overall health of the individual while those adopting the modern subluxation theory hold that the subluxation has a more local biomechanical affect. It is often reported that TPS practitioners represent only a small portion of the overall profession [80]. This is not the case. A survey of North American chiropractors found amongst respondents, in excess of 75% of those adhering to the modern subluxation construct believe that the adjustment of the vertebral subluxation complex usually elicits improvements in select visceral ailments [81]. Compounding the problem is the commonly relayed message that subluxations are only detectable by the skilled hands of a qualified chiropractor. Given this, one must ask: what is the evidence supporting the TPS ideology that minor vertebral displacements cause visceral disease?

Nansel and Szlazak [82] carefully examined this very question and concluded scientific consensus does not support the theory that nerve interference caused by vertebral misalignment or subluxation is a cause of visceral disease. Further, Nansel and Szlazak clearly state “there have been no appropriately controlled studies that establish that spinal manipulation or any other form of somatic therapy [manual therapy] represents a valid curative strategy for the treatment of any internal organ disease”. It is noteworthy that Nansel and Szlazak’s findings have never been challenged.

Worse yet, from an epidemiological perspective (strength of association, specificity, temporality, dose response, experimental evidence, biological plausibility, coherence and analogy), the subluxation is found to be wanting [83] as is adherence to subluxation theory as a mode of practice [70, 84–89].

Many chiropractors argue for the ‘neurological effects’ of the adjustment. While it is safe to say that the high velocity low amplitude thrust employed by chiropractors has a neurological effect, as does stubbing one’s toe, it is a stretch to suggest that the adjustment’s effect is predictable, lasting and always positive. Haldeman, a qualified chiropractor, medical neurologist and holder of a PhD in neurology, wrote on the subject of the purported neurologic effects of the chiropractic adjustment.

A significant leap of faith is required to accept and present a convincing argument about the various theories on the neurologic effects of the adjustment. …

What must be avoided at this stage of understanding of the neurologic effects of the adjustment is the unreasonable extrapolation of current knowledge into speculation and presentation of theory as fac [90].

Thus we have a large portion of the chiropractic profession, a group recognized as being the third largest primary-contact health care profession in the Western world [80], telling health care consumers that undetected subluxations, if not removed, will result in adverse health consequences and an untimely death. This is a position unsupported by evidence. In short, it is an unsubstantiated claim the purpose of which is to drive asymptomatic members of the public into chiropractor’s offices to receive examinations to locate subluxations and treatment to remove killer subluxations before they cause unimaginable harm to the individual. Undeniably, it is the appeal to fear fallacy exemplified.

Not only is it unethical [88] it is totally inappropriate. Some chiropractors have even gone so far as to suggest that subluxation based practice is a threat to the public health [91]. Given the best available evidence I suspect chiropractors marketing their care in this fashion could be faced with the unenviable task of defending against an allegation of unconscionable conduct under Australian Consumer Law.

## Conclusion

I have briefly examined the appeal to fear as it is used in mainstream health care and established that for the most part its appropriate use is effective and not fallacious. It is however used fallaciously by a large element within the chiropractic profession in its marketing of the unsubstantiated effects of chiropractic subluxation.

Even though most people realise that the sky is not falling, gullible Chicken Little types still abound, particularly amongst vulnerable health care seekers. Most rational chiropractors realise that the sky is not falling and that subluxations are not a health risk. That said, the evidence presented is clear: appeal to fear marketing is alive in chiro-land.

The data demonstrates that appeal to fear as a marketing tool is of dubious value. After taking into consideration the best available evidence the conclusion is clear: the appeal to fear when used judiciously in some health care settings is acceptable. These settings include anti-smoking, anti-drunk driving and hypertension awareness campaigns.

Use of appeal to fear is totally unacceptable and fallacious when used to promote health care interventions for which there is no credible evidence such as chiropractic subluxation based care. Attempts to convince the unsuspecting public that subluxations present a clear and present danger is a falsehood at best and unconscionable conduct at worst.

It is well beyond time that the authorities governing the chiropractic profession, namely chiropractic registration boards as well as those advocating for chiropractic (the professional associations) take the appropriate steps to eliminate the appeal to fear from chiropractic marketing.

## Footnotes

1 A compilation of the 40 anti-smoking television ads judged to evoke the greatest ‘fear’ response can be found at: https://helloimapizza.com/2014/12/07/smoking/

2 People driving under the influence of alcohol are commonly referred to as drunk drivers, or drink-drivers depending on the jurisdiction. To avoid confusion, drunk driving will be the term used throughout this paper.
